# Endoscopic Preoperative Assessment, Classification of Stenosis, Decision-Making

**DOI:** 10.3389/fped.2019.00532

**Published:** 2020-01-08

**Authors:** Marta Filauro, Francesco Mazzola, Francesco Missale, Frank Rikki Canevari, Giorgio Peretti

**Affiliations:** ^1^IRCCS Ospedale Policlinico San Martino, Genoa, Italy; ^2^Unit of Otorhinolaryngology—Head and Neck Surgery, University of Genoa, Genoa, Italy; ^3^Department of Molecular and Translational Medicine, University of Brescia, Brescia, Italy

**Keywords:** classification, laryngotracheal stenosis, decision-making, pediatric airway, European laryngological society

## Abstract

Pediatric Laryngo-Tracheal Stenosis (LTS) comprises different conditions that require precise preoperative assessment and classification. According to the guidelines, the optimal diagnostic work-up of LTS patients relies both on a comprehensive anamnesis and on endoscopic and radiological assessments. All the causes of LTS result in an impairment in airflow, mucociliary clearance, phonation, and sometimes in swallowing disorders. The main goals of treatment are to maintain an adequate respiratory space and restore the Upper Aero-Digestive Tract (UADT) physiology. The first step when dealing with LTS patients is to properly assess their medical history. The main causes of pediatric LTS can be divided into two groups, i.e., congenital and acquired. The most common causes of congenital LTS are: laryngomalacia (60%), vocal fold paralysis (15–20%), subglottic stenosis (SGS) (10–15%), laryngeal webs and atresia (5%), subglottic hemangioma (1.5–3%), and others. On the other hand, 90% of acquired pediatric LTS cases are subsequent to post-intubation injuries. Other less frequent causes are: iatrogenic complications from endoscopic laryngeal interventions, benign tumors, caustic or thermal injuries, external blunt force injury or trauma, chronic inflammatory disorders, or idiopathic causes. Diagnostic work-up consists in a step-by-step investigation: awake and asleep transnasal fiberoptic laryngoscopy (TNFL), direct laryngoscopy with 0° and angled telescopes to investigate the type of stenosis (arytenoid mobility, craniocaudal extension, involved anatomical sites, and active or mature scar tissue), and broncho-esophagoscopy to rule out associated mediastinal malformations. To date there are several available classifications for each of the involved sites: Cohen's classification for anterior glottic stenosis, Bogdasarian's for posterior glottic stenosis (PGS) and Myer-Cotton's for subglottic stenosis, even though others are used in daily practice (Lano-Netterville, FLECS, etc.). The European Laryngological Society recently proposed a new classification which is applicable in all LTS cases. In this chapter we deal with preoperative assessment and staging, reviewing the most relevant classifications applicable in patients affected by LTS, *conditio sine qua non* in order to tailor the best treatment modality to each subject. We'll also detail the comprehensive radiological, endoscopic and functional assessment for the correct use of each staging classification.

## Introduction

Pediatric laryngotracheal stenosis (LTS) comprises a wide number of conditions that require precise pre- and intraoperative assessment.

The main causes of pediatric LTS can be divided into two groups, i.e., congenital and acquired. The most common causes of congenital LTS include laryngomalacia (60%), vocal fold paralysis (15–20%), subglottic stenosis (SGS) (10–15%), laryngeal webs and atresia (5%), subglottic hemangioma (1.5–3%), and others (1). On the other hand, 90% of acquired pediatric LTSs occur after post-intubation injuries. Other less frequent causes include iatrogenic complications from endoscopic laryngeal interventions, benign tumors, caustic or thermal injuries, external blunt force injury or trauma, chronic inflammatory disorders or idiopathic causes (2).

The aims of the initial assessment are to establish or confirm the diagnosis, to identify disease-specific risk factors and prognostic variables, to set goals with the patient and his/her parents and to establish an initial management plan (3).

Accurate preoperative and intraoperative diagnostic work-up is of paramount importance to obtain crucial information which could impact on the postoperative outcome. Information that must be collected includes vocal fold mobility, the presence of glottic and/or supraglottic scar tissue, crico-arytenoid joint(s) fixation, possible additional tracheal damage (stenosis, malacia) related to stoma or cannula, secondary airway lesions (i.e., granuloma, scar tissue), obstructive sleep apnea (OSA)-related obstructions, swallowing difficulties with/without chronic aspiration, severe gastroesophageal reflux (GOR), eosinophilic esophagitis, medical comorbidities, or congenital anomalies.

Any of the following scenarios may be encountered in a child with acquired LTS: (1) neonatal intubation for respiratory distress; (2) intubation for infection or traumatic injury later in life; (3) previous history of endotracheal intubation presenting as “idiopathic” LTS (4).

An important distinction that must be pointed out is between incipient and mature stenosis. Incipient LTS results from acute or subacute post-intubation airway narrowing (e.g., edema, ulcerations, granulation tissue), which is treated endoscopically or by a cricoid split procedure in newborns in an effort to prevent cicatricial stenosis. The final goal is to avoid tracheotomy or allow decannulation in already tracheostomized patients (2).

Mature cicatricial stenoses correspond to well-established airway narrowing that can pose a therapeutic challenge to the surgeon. It is thus of paramount importance to understand the individual characteristics of the stenosis and the clinical context of each patient (5).

In order to identify the best therapeutic option, an appropriate diagnostic work-up must include correct staging of the lesion and an endoscopic pre- and intraoperative assessment.

## Classifications of LTS

In the last few decades, the broad variety of laryngotracheal stenosis presentations has been in great need of standardized definitions. In an attempt to fill this gap, laryngotracheal stenosis classifications have proliferated in the literature due to the need to describe the complex anatomy of this district, the varied clinical presentations and the treatment possibilities. The most relevant classifications are reported in this chapter.

### Myer-Cotton

**Figure 1:**
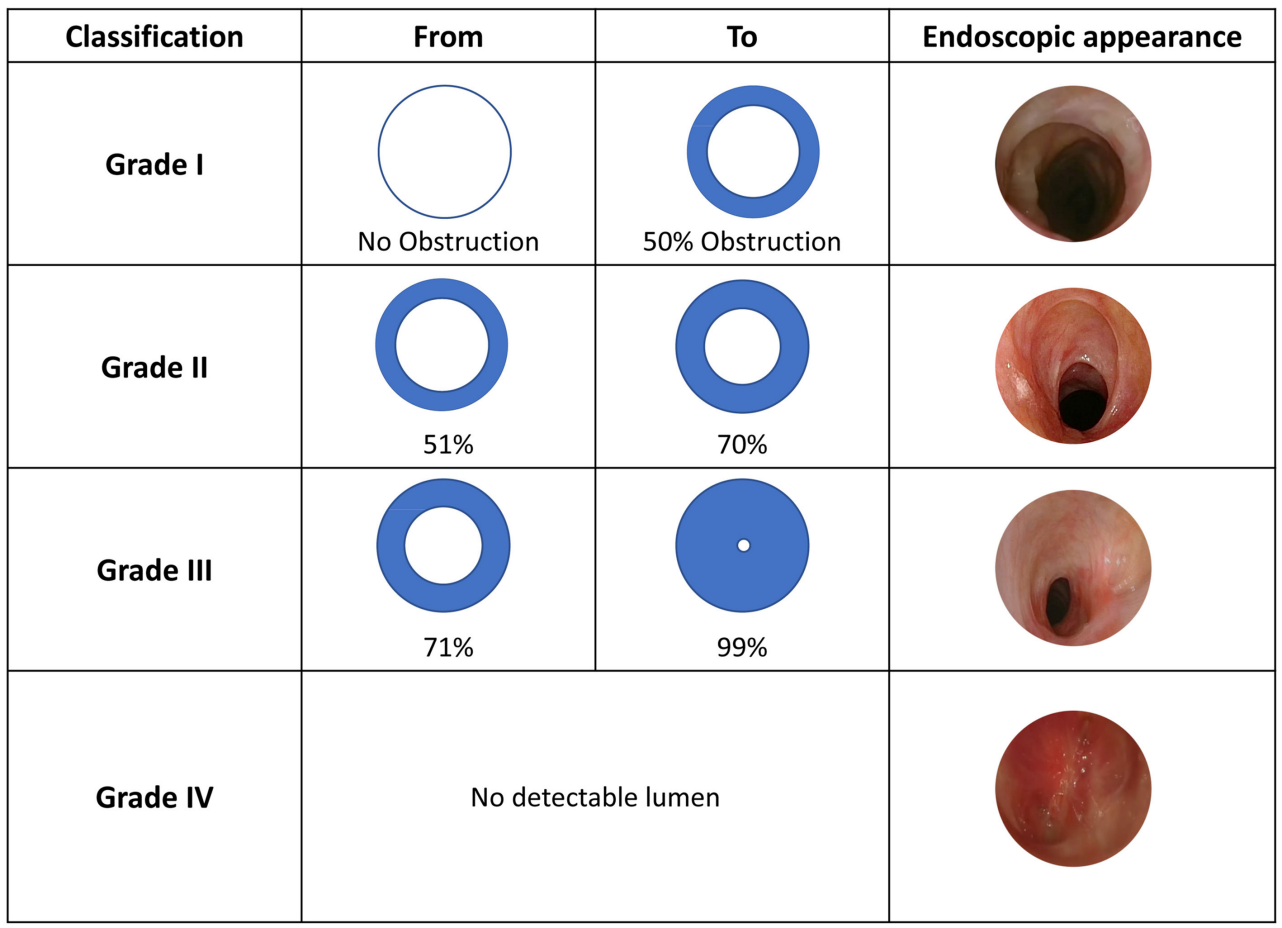
Myer-Cotton classification, adapted from Myer et al. (3) with unpublished clinical pictures.

This classification was proposed by Myer et al. (3), taking cues from a previous version published by Cotton (4). The Myer-Cotton classification was intended for firm, mature subglottis stenoses, thus excluding any other lumen narrowing conditions (e.g., trachea- and/or laryngomalacia, vocal cord paralysis, immature stenosis, tracheal stenosis, suprastomal collapse, supraglottic collapse and suprastomal granulation tissue). While it was initially used to predict lumen surface reduction in case of endotracheal tube application, it was then extended to describe both pediatric and adult subglottic and/or tracheal stenosis. It consists of IV grades: Grade I−0 to 50% decrease in lumen surface; Grade II−51 to 70% decrease; Grade III−71 to 99% decrease, and Grade IV–no evidence of detectable lumen (Figure 1). Healthy and stenotic lumen surfaces can be accurately calculated and compared by radiological imaging or intra-operative investigation using the formula A = πr^2^ to obtain circle area values.

### McCaffrey

**Figure 2:**
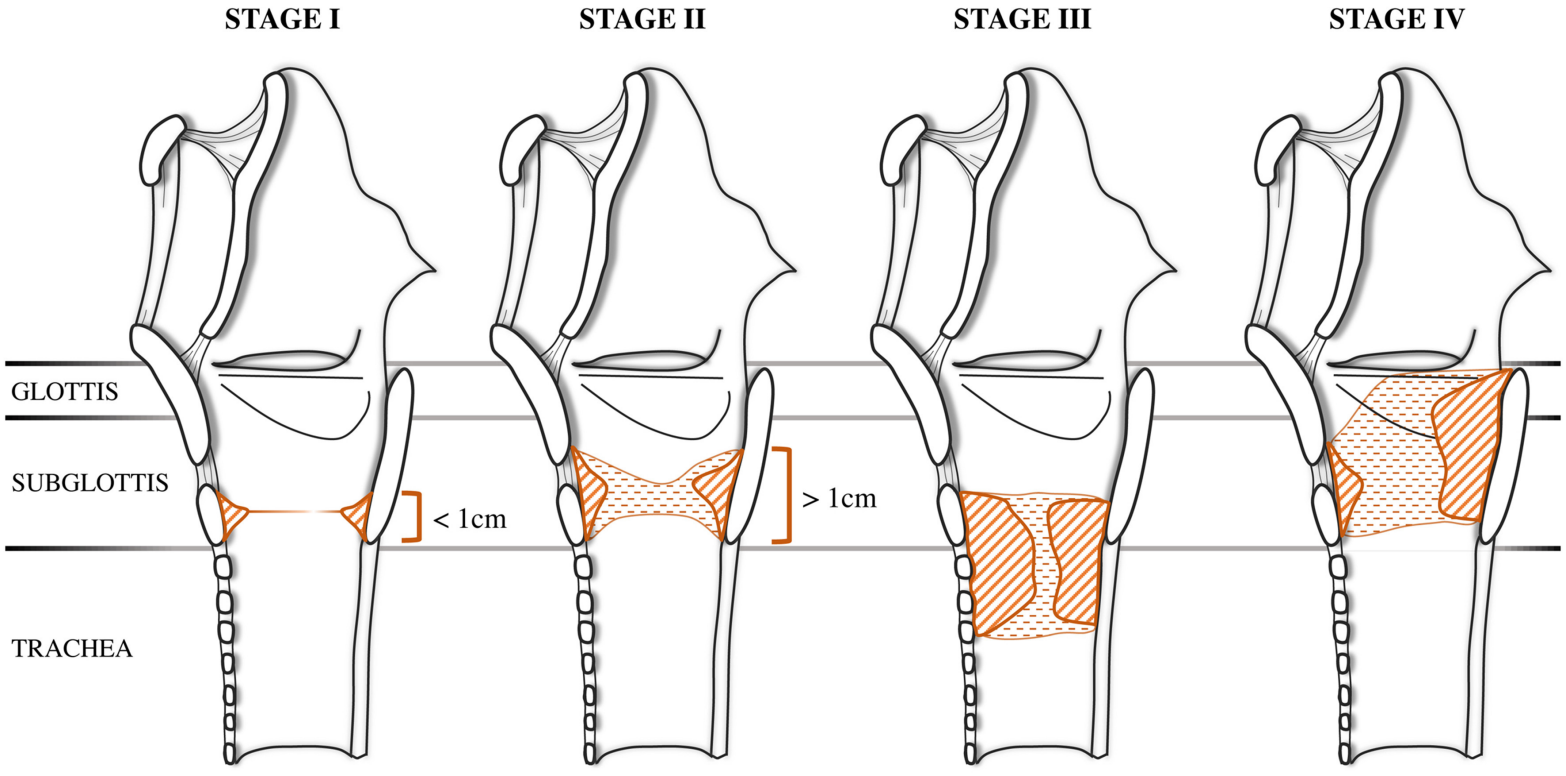
McCaffrey Classification. The stenosis can be located in the subglottis or in the trachea in stage I and in stage II, adapted from McCaffrey (6) with unpublished draws.

In 1992, McCaffrey proposed a classification to describe the extension of the stenosis among the most commonly involved subsites: glottis, subglottis and trachea (6). This system was initially validated on an adult population but was later extended to pediatric patients. This classification was conceived after both univariate and multivariate analyses showed that the involved site had the most consistent significant predictive value for determining time to decannulation. Stage I is located in the subglottis or trachea, with a craniocaudal extension of <1 cm. Stage II stenosis is limited to the subglottis and has a craniocaudal extension of more than 1 cm. Stage III stenosis involves both the subglottis and trachea, while Stage IV stenosis extends to the glottis with fixation or paralysis of at least one vocal cord (Figure 2).

### Lano-Netterville

In 1998, Lano et al. (7) proposed a simpler staging system based on anatomical subsite involvement. The authors described three stages according to the number of involved subsites: Stage I in case of single subsite involvement, stage II for two involved subsites and stage III in case of glottic-tracheal involvement (Table 1). The Authors recommended the use of this classification in order to have a good correlation between surgical success and stage categories, which in their retrospective population resulted 94, 78, and 20% for Stage I, II, and III, respectively.

**Table 1:** Lano-Netterville classification Lano et al. (7).

	**N. of involved subsites**	**Subsites**
Stage I	1	Glottis or sub-glottis or trachea
Stage II	2	Glottis and sub-glottis or sub-glottis and trachea
Stage III	3	Glottis and sub-glottis and trachea

### Cohen

**Figure 3:**
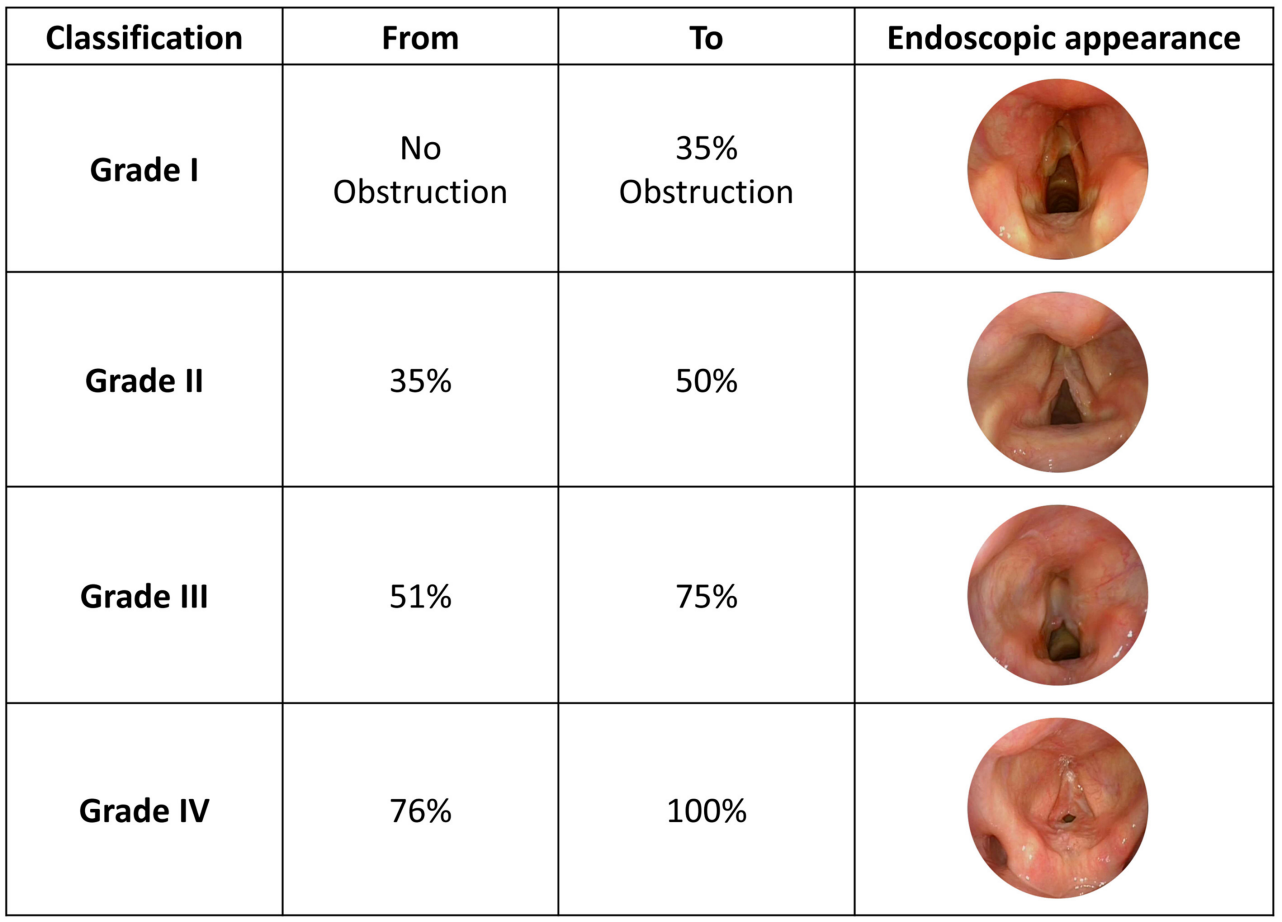
Cohen Classification, adapted from Monnier et al. (5) with unpublished clinical pictures.

A new classification proposed by Cohen (8) attempted to describe anterior glottic webs occurring at the time of glottic lumen formation during laryngeal embryological development (1985). The four types of anterior glottic web correlate with the severity of symptoms. Type 1 is an anterior web involving 35% or less of the glottic lumen. Type 2 ranges between 35 and 50%, Type 3 between 51 and 75% while Type 4 involves 76% or more of the glottic lumen (Figure 3).

### Other Classifications

As previously mentioned, other classifications have been proposed, and while each of them describes stenotic lesions differently, they have limited relevance in clinical practice.

Laccourrey et al. (9) proposed a detailed staging system taking into account larynx fixation, subsites, and laryngeal joint involvement.

Bogdasarian et al. (10) published a new classification to describe post-intubation posterior glottic stenosis (PGS) in the pediatric population. Moreover, the FLECS staging system (11) was proposed by the American Society of Pediatric Otolaryngology (ASPO) to encompass a detailed description of the Function, Lumen, Diameter, Extent and Site of stenosis. Although this classification was probably the most complete, it was abandoned because it was considered too confusing.

With regard to laryngomalacia, the most widely used classification was proposed by Olney et al. (12). Type 1 corresponds to a prolapse of the mucosa overlying the arytenoid cartilage, type 2 to foreshortened aryepiglottic folds and type 3 to posterior displacement of the epiglottis.

### ELS Classification

In 2015, the European Laryngological Society (ELS) published a consensus paper (5) presenting a five-step endoscopic airway assessment and a reporting system. The authors attempted to standardize the pre- and intraoperative assessment techniques in order to fully investigate the lesion in all its aspects (type of stenosis, involved subsites, extension, laryngeal motility, and airway lumen). Furthermore, other conditions describing airway pathological status or concurrent comorbidities were integrated to help provide a more comprehensive evaluation of the patient. All these data are enclosed in a practical checklist which generates a final score.

The ELS classifications has been applied to large retrospective cohorts from the centers that contributed to its developing and it was confirmed to be as accurate for the prediction of both peri-operative outcome, i.e., complication rate or need for further treatments as long as for long-term outcomes, i.e., decannulation rate, both in pediatric (13) and adult groups (14) affected by laryngotracheal stenosis, and treated by tracheal or crico-tracheal resection and anastomosis. Its value for the prediction of the outcomes of patients submitted to endoscopic treatments has not been assessed yet.

The endoscopic work-up for LTS according to the ELS consists of five key-point procedures:

1. Awake indirect laryngoscopy or transnasal fiberoptic laryngoscopy (TNFL);
2. Asleep TNFL;
3. Direct transoral laryngotracheoscopy with a bare zero-degree rod-lens telescope;
4. Suspension microlaryngoscopy;
5. Bronchoesophagoscopy;

See the next paragraph for a detailed description of each step (5).

### Radiological Assessment

Unlike indications for radiological evaluation in adult patients, those for pediatric stenosis are still under debate. CT scan can be helpful for studying craniocaudal extension and severity of the obstruction, capable to provide also 3D reconstruction of the airways (15, 16), especially when awake TNFL is unable to show a clear view of them. Indeed, before performing any procedure under sedation or general anesthesia, radiological investigation is always mandatory in patients with Cotton-Meyer grade III or IV stenosis, except for subjects requiring immediate tracheotomy. Good quality imaging can be obtained without sedation using a CT scan with ultrafast acquisition frames. Otherwise, radiological imaging is indicated to study neck masses or abnormal mediastinal vessels (17). If the child can be safely sedated, standard CT or MRI scans can be performed in the presence of an anesthetist. MRI provides high resolution images allowing the assessment of airway compression secondary to mediastinal malformations (5).

### Functional Assessment

Voice evaluation and respiratory function assessment should be obtained before carrying out any conservative or open neck procedures in order to establish the baseline condition. Documentation regarding stridor at-rest or during exercise, and physical activity and pulmonary function tests, such as spirometry, must be reported (18). Moreover, voice evaluation should be performed using the GRBAS-scale, including voice range profile and maximum phonation time for the vowels/e/and/a/. In case of infant patients these clinical data are meaningless if the tests cannot be performed properly (5).

### Assessment of the Patient's General Condition

A comprehensive assessment of concomitant comorbidities must be included in the work-up. Pulmonary and cardiac check-ups are required, together with a full neurological evaluation. In case of previous tracheostomy, the grade of stenosis can easily be tested by temporarily occluding the cannula. This test provides immediate feedback on pulmonary capacity and low-flow/high-pressure resistance of the stenotic area (5).

### Final Scoring

ELS final scoring is obtained by combining the Myer-Cotton grading system, a revised version of the Lano-Netterville classification, and the presence of severe comorbidities. The Myer-Cotton score defines the degree of stenosis and, similarly to the original classification, assigns a value from I to IV.

**Figure 4:**
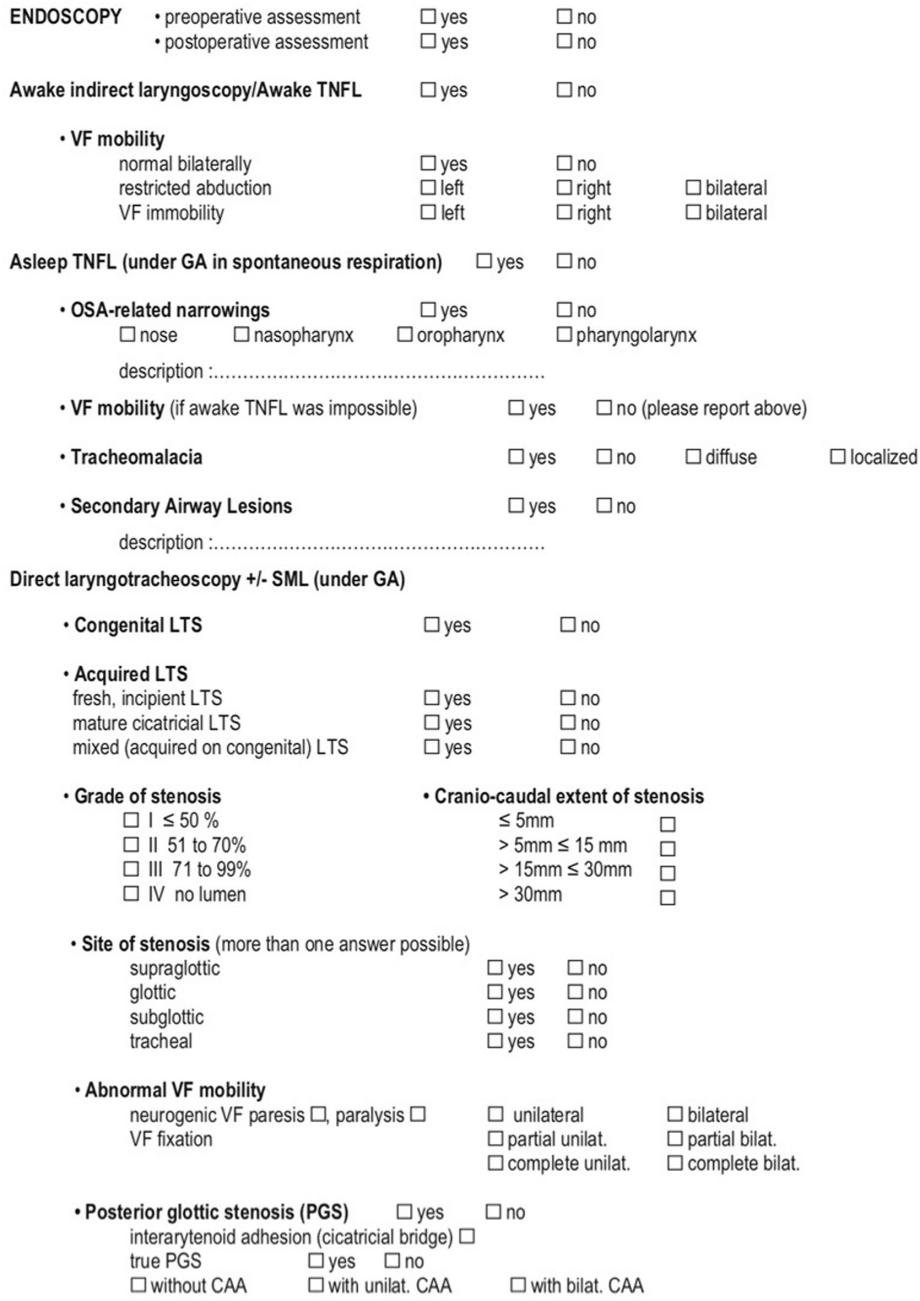
ELS checklist for pre-operative assessment and decision making (1/2) Monnier et al. (5).

**Figure 5:**
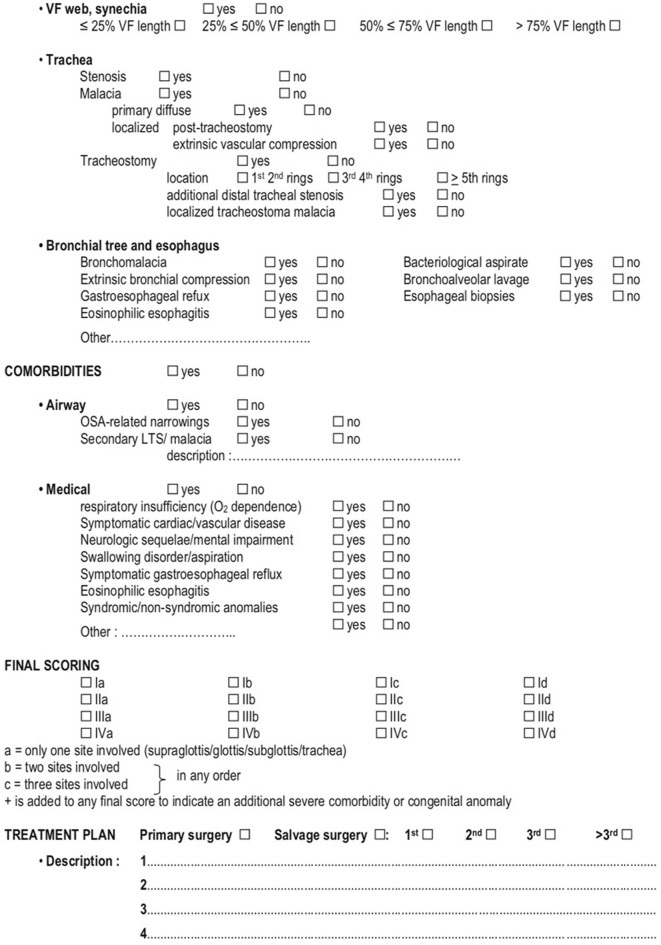
ELS checklist for pre-operative assessment and decision making (2/2) Monnier et al. (5).

Thereafter, a revised version of the Lano-Netterville classification, which includes the supraglottis as the fourth subsite, is applied: (a) establishes a single subsite involvement and so forth up to (d) for involvement of all subsites. Lastly, in case of severe comorbidity or congenital abnormalities, a (+) sign is added to the score (Figures 4, 5) (5).

### Advantages and Limits of ELS Classification

The main advantage of ELS classification is its comprehensive value and its aid guiding the whole work-up for a patient affected by laryngotracheal stenosis; regarding the scoring system it introduces the three-dimensional view, taking into account together the cranio-caudal shape of the stenosis and its smaller area on the axial plane; furthermore, relevant comorbidities of the patient are included, as they are crucial for the correct decision-making process. Beyond all these favorable features of the ELS classification, it has some limits as it lacks specificity for some subsites of the airway, such as the different compartments of the glottis, for which the older Cohen's and Bogdasarian's classification still play a relevant role for the staging of anterior and posterior glottic stenosis, respectively. Secondary, the functional investigation, such as spirometry, is included in the suggested work-up but its specific parameters are not taken into consideration in detail; beneath this kind of test is mostly applicable in adults population, the results by the recent literature about the usefulness of such exam are rising the value of spirometry for an objective evaluation of patients both for the pre-treatment staging and for follow-up period (19, 20).

## Preoperative Endoscopic Assessment

The endoscopic diagnostic work-up includes TNFL, asleep TNFL, direct transoral laryngoscopy with 0° and 70° telescope, suspension microlaryngoscopy (SML), and bronchoesophagoscopy. Upon completion of the endoscopic work-up, a radiological study with CT and/or MRI must be performed, as must an assessment of respiratory and voice function and of the patient's general conditions.

### Transnasal Fiberoptic Laryngoscopy

TNFL is part of the in-office examination. Patency of the nasal cavities, choanae, nasopharynx, pharynx, and larynx should be carefully assessed.

Flexible nasoendoscopy is performed to detect any obstructive supraglottic lesions (e.g., laryngomalacia, lymphovascular malformations, and cysts), vocal fold movements, and any evidence of impaired swallowing such as hypopharyngeal pooling of secretions. If restricted abduction or true immobility is observed, then a complete examination under general anesthesia is warranted in order to distinguish neurogenic bilateral vocal cord paralysis from posterior glottic stenosis, especially if the patient has been subjective to an orotracheal intubation. TNFL always yields incomplete information as the subglottis and trachea cannot be properly visualized.

Not all patients need direct laryngoscopy, especially when a clear diagnosis of mild laryngomalacia is made following a comprehensive medical history and physical examination (21). However, in case of symptom worsening, associated abnormalities or atypical clinical presentations, a complete laryngotracheobronchoscopy and esophagoscopy must be performed under general anesthesia.

### Asleep TNF

Clinical indicators such as feeding difficulties, growth slowdown, obstructive sleep apneas and pulmonary hypertension required further investigation. Moreover, inadequate patient compliance is an absolute indication for TNFL under general anesthesia but in spontaneous respiration. Anesthesia is maintained with sevoflurane or i.v propofol. Before starting the laryngoscopy, atropine is administered intravenously.

Inspection of the nasal cavities on both sides is mandatory to identify possible pathologies, such as vestibular stenosis, deviated septum, choanal atresia, adenoid hyperplasia, or tumor masses. In cases of obstructive sleep apnea, when patients undergo general anesthesia with spontaneous breathing, their muscle tone decreases and the level of obstruction can then be identified. Various causes of dynamic obstruction that are detectable by fiberoptic endoscopy include retroposition of the soft palate, lingual tonsillar hypertrophy and epiglottic and supraglottic prolapse. This assessment is extremely relevant, especially in the preoperative evaluation of subglottic stenosis. A jaw lift is mandatory to raise the tongue base, allowing a better visualization of the pharyngolarynx. When the video-bronchoscope reaches the laryngeal aditus, deeper anesthesia is needed in order to further advance into the lower airway tract without risking laryngospasm.

Passing behind the epiglottis and reaching the laryngeal inlet allows for a detailed and careful assessment of vocal cord mobility (22): it should be noted that large cuneiform cartilage and short aryepiglottic folds can obscure the laryngeal inlet and prevent a proper view of the vocal cords. Flexible laryngoscopy in the office setting might not be able to identify oropharyngolaryngeal obstructions that may be responsible for OSA.

Evaluation of the larynx during spontaneous breathing allows us to identify webs, posterior glottic stenosis, vocal fold palsy or paralysis.

Furthermore, dynamic examination of the trachea and bronchi is of paramount importance for the diagnosis of localized or diffuse tracheomalacia and anatomical narrowings of the lower airways. When vocal cord immobility is detected during TNFL, SML must be carried out.

In conclusion we can say that both awake and asleep TNFL are required in the evaluation of a compromised airway.

#### Direct Transoral Laryngoscopy With 0° and 70° Telescope

In order to better visualize and characterize a possible glottic, subglottic or tracheal stenosis the child must be deeply anesthetized and completely paralyzed. The larynx is visualized using a general-purpose Storz laryngoscope with the blade inserted at the level of the vallecula. A rigid 4 mm diameter magnifying telescope offers a clear view of the endolarynx, subglottis and trachea as far as the carena. In case of subglottic or tracheal stenosis, attention has to be paid not to damage the mucosa. Indeed, the slightest injury to a narrow airway could decompensate a stable obstructive dyspnea, thus requiring a tracheotomy. If the 4 mm diameter endoscope is too large, then a 2.7 mm or even a 1 mm diameter (sialendoscopy) scope should be used to assess the length of the stenosis and the integrity of the distal airway. The degree of SGS is calculated by passing telescopes or bougies of different sizes through the stenosis. The Myer–Cotton airway grading system is commonly utilized (3). Generally speaking, a tracheostomy caused by diagnostic upper airway endoscopy must be considered unacceptable.

### Suspension Microlaryngoscopy

The Benjamin-Lindholm laryngoscope is preferable for visualizing the pharynx, larynx, and subglottis (23).

Specific tools are needed during the procedure: 0° and 70° telescopes, a Lindholm vocal cord retractor, angulated probes, and tapered bougies.

**Figure 6:**
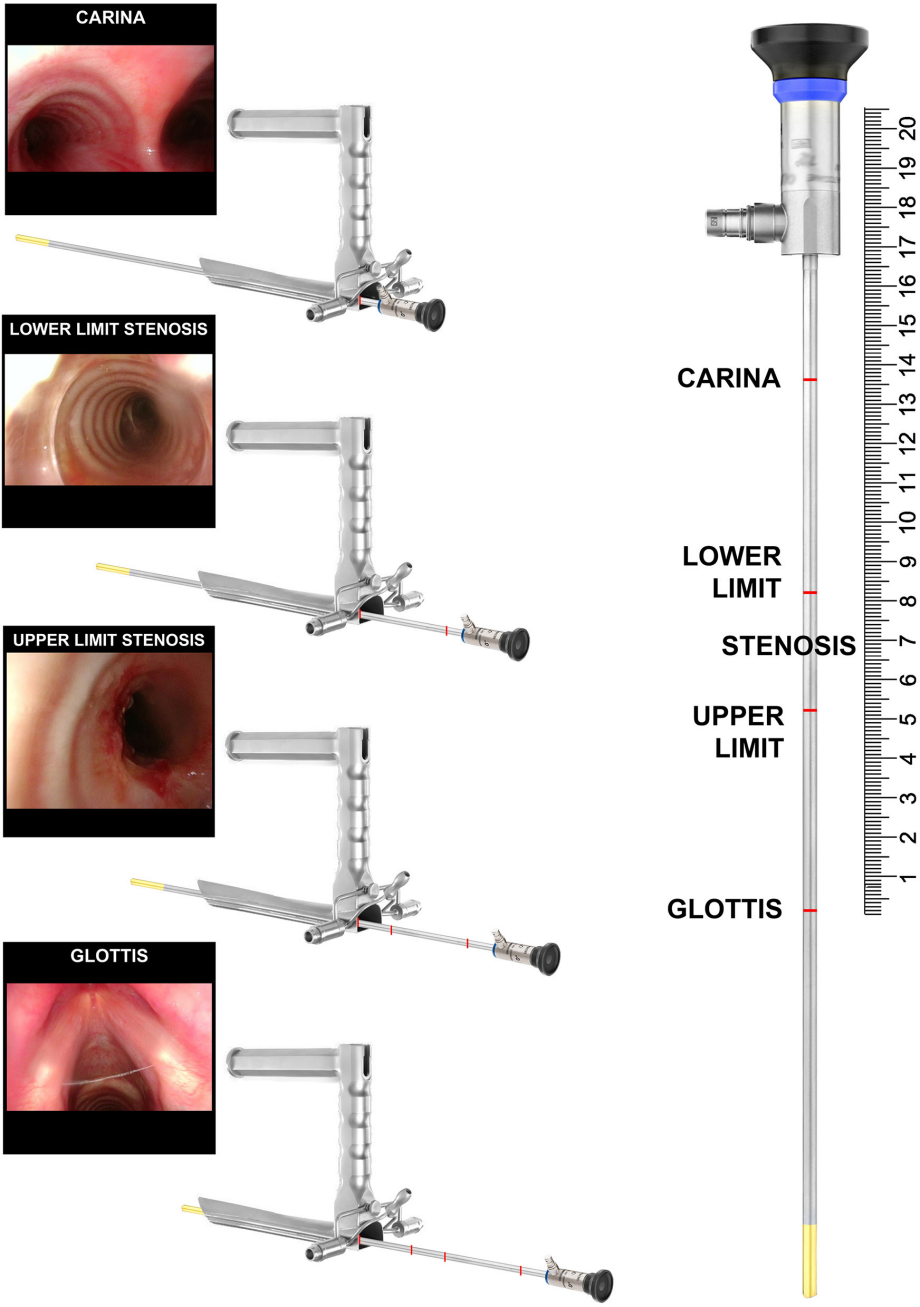
Intraoperative measurement of a 3 cm tract of the tracheal stenosis during suspension microlaryngoscopy.

Telescopes are used to measure the craniocaudal extension of the stenosis. Bougies of given size are used to measure the degree of the stricture. Lastly, in order to distinguish between bilateral vocal cord paralysis (BVCP) and posterior glottic stenosis, Lindholm vocal cord retractor and angulated telescopes are used. To get the most precise measurement of the craniocaudal extension of the stenosis, the telescope is inserted through the laryngoscope and further advanced as far as the vocal cords, pointing the distance on the shaft of the telescope. Repeated measurements are taken at the upper and lower borders of the stenosis and tracheostoma, if present, and lastly at the level of the carina (Figure 6). In order to program the surgery correctly, especially in case of tracheal resection and anastomosis, such measurements are mandatory. With complete airway obstruction, CT scans with 3D reconstructions are very useful.

**Figure 7:**
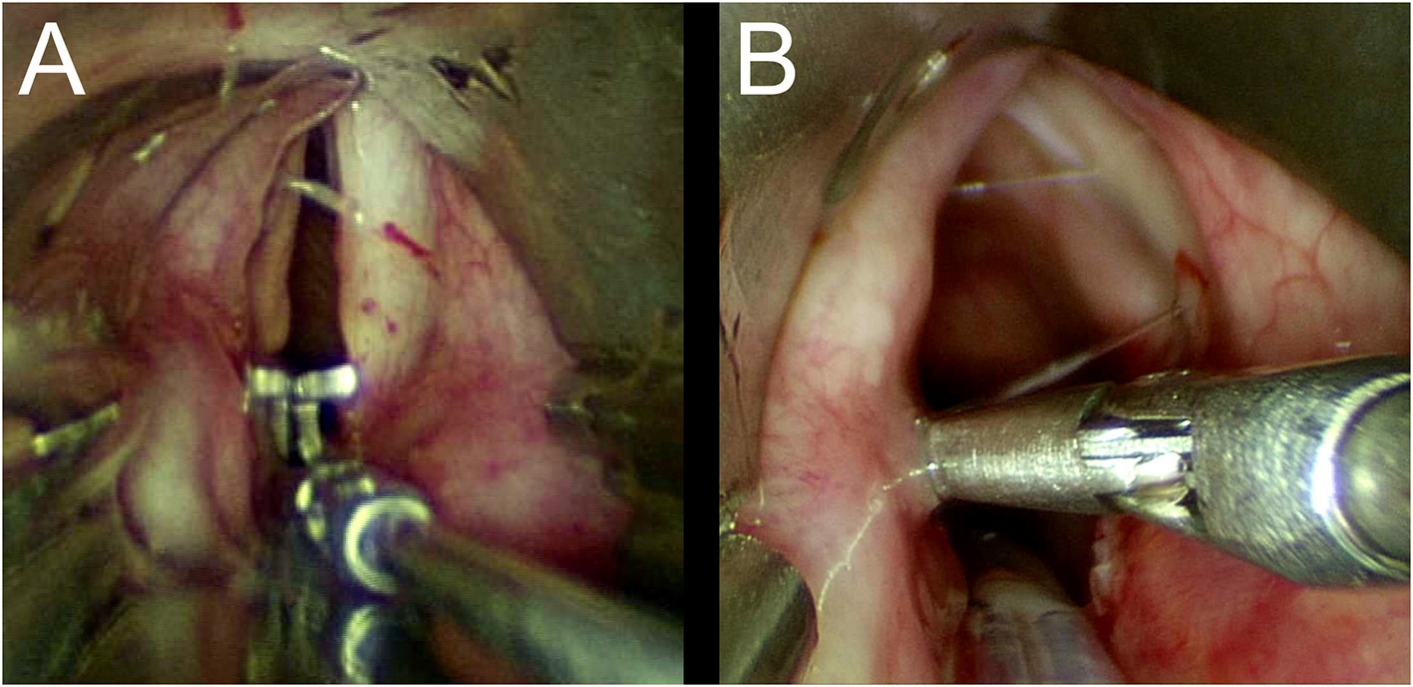
**(A,B)** Lindholm cord retractor.

Once we had carefully assed the craniocaudal extension and the degree of the stenosis, differential diagnosis between BVCP and PGS is mandatory. Important information can be provided by the patient's medical history. Lindholm cord retractor and an angulated probe are used to precisely assess the posterior laryngeal commissure and cricoarytenoid joint function in all the patients who have previously undergone orotracheal intubation. The former is placed at the level of the vocal fold and is opened. In patients with neurogenic BVCP, the interarytenoid distance is restored to its normal size, while in patients with PGS it remains narrow, and a stretched band of scar tissue may be observed (Figures 7A,B).

**Figure 8:**
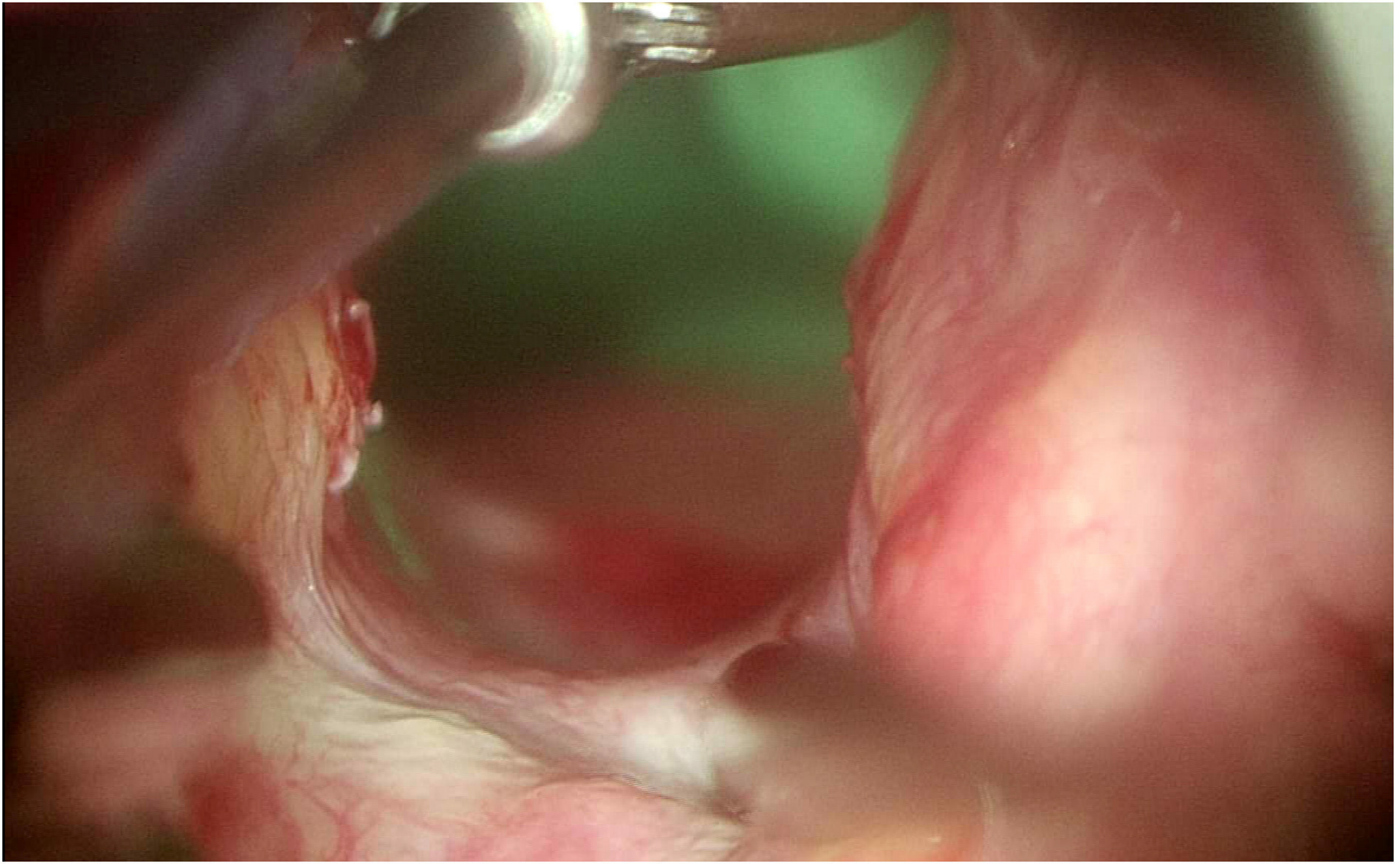
Angulated probe to better visualize the posterior commissure.

By using the angulated probe, it thus becomes possible to precisely identify the different types of PGS according to Bogdasarian's classification (Figure 8) (10).

### Bronchoesophagoscopy

The diagnostic work-up of children affected by SGS must be completed by the evaluation of lower airways and esophagus. In case of tracheostomized patients, rigid or flexible bronchoscope has to be introduced through the tracheostoma. If the distal end of the cannula has caused strictures of the lower trachea, the rigid bronchoscope is not advanced any further in order to avoid mucosal damages. In this case the use of a flexible bronchoscope is mandatory. If the tracheal wall has not been damaged by the cannula, then all of the rings can be identified. The linear measure between the lower border of the tracheostoma and the carina should be measured as described in the previous paragraph (Figure 6). When planning possible resection and anastomosis of the airway it is of paramount importance to count the number of residual normal tracheal rings. Further investigation down to the basal bronchi is performed on both sides.

Biopsies and bronchoalveolar lavage (BAL) should always be executed at the end of bronchoscopic evaluation, as bleeding might affect any further examination.

During the bronchoscopy we have to investigate the presence of congenital or acquired lesions such as tracheo-esophageal fistula, bronchus suis, localized or diffuse malacia, extrinsic compressions, anomalous distribution of the bronchial tree and lesions caused by local trauma induced by the tracheostomy cannula as well as from suction catheters at the level of the carina. The characteristics of bronchial secretions (serous, mucus, muco-purulent) and mucosa (inflammation and friability) must be documented. Precise assessment of their effects on the ventilation through the stenotic segmental bronchi is required. Bacteriological testing of the aspirate should be conducted systematically, while BAL is necessary to confirm the diagnosis of chronic aspiration when lipid-laden macrophages are seen on the smear examination. Missing the diagnose of infection of the lower airways could affect surgical outcome, leading to adverse complications such as anastomotic dehiscence, cartilage graft infection and secondary tracheostomy.

As well as bronchosocopy, esophagoscopy can be performed with flexible or rigid scopes. The use of rigid scope is easier in infants and children than in adults (24, 25).

Esophagoscopy must address the presence of gastroesophageal reflux and eosinophilic esophagitis.

GOR is best diagnosed using 24-h pH monitoring or impedancemetry, but endoscopy may reveal signs of erosive esophagitis (25). The absence of the angle of His, with the cardia opening located in a direct line to the gastric pouch, is an anatomical pattern that may cause chronic reflux.

Thickened or annulated mucosa may be indicative of eosinophilic esophagitis (26). Biopsies are necessary to confirm this diagnosis.

## Decision Making

Comprehensive pre-operative assessment is helpful for identifying which children affected by laryngotracheal stenosis would most benefit from surgery. It would also be useful to the multi-disciplinary laryngotracheal stenosis board team made up of ENT surgeons, pulmonologists, gastroenterologists, cardiologists, neurologists, neonatologists, and intensivists in making the best choices regarding type and timing of surgery (2). The goal of any laryngotracheal surgical procedure is to achieve decannulation and to re-establish an airway while preserving adequate laryngeal function for airway protection, swallowing, and voicing (27).

### Tracheotomy

Patients affected by mild stenosis (grade I-II) are often kept under observation due to the lack of severe symptoms and pending airway growth that usually leads to widening of the free lumen. If the patient's respiratory condition starts deteriorating it is advisable to take airway reconstruction surgery into consideration to avoid a tracheotomy, the latter making further treatment more challenging and being a potential worsening factor for learning effective communication in a growing child.

In some cases, however, tracheotomy is unavoidable in order to safely secure a compromised laryngeal airway, especially in the presence of local or systemic medical conditions which are contraindicated in airway reconstruction surgery (28). It must be highlighted that the mortality rate of tracheostomized children with significant laryngeal obstruction has decreased from 24% in the 1970s to 2–3% by the year 2000 (2). When dealing with a laryngeal stenosis, the tracheotomy should be placed immediately below the cricoid to maximally preserve the normal trachea distally, or far below, sparing a sufficient amount of uninvolved tracheal rings between the stenosis and the tracheostomy. With this aim in mind, the best site to perform the procedure in patients with purely tracheal stenosis is through the stenotic site, maximally preserving the uninvolved tracheal rings.

### Surgical Timing

The main factors affecting the choice to proceed to surgery primarily regard open neck laryngo-tracheal reconstruction procedures, meaning partial cricotracheal resection and anastomosis (PCTR) or laryngotracheal reconstruction (LTR), and can be summarized in three main items: grade of stenosis, inflammatory status of the stenosis and/or medical comorbidities, age and weight of the child.

1. Grade: for children affected by mild stenosis (Grade I-II) a wait and see policy can be chosen, especially facing with congenital, knowing that the airway is expected to become wider with growth. Endoscopic follow-up should be done every 6 months, until complete resolve of the symptoms. If a worsening of the respiratory condition is seen, an airway reconstruction surgery must be taken into account, to avoid emergency tracheostomy, potentially compromising further airway reconstructive procedures (2);
2. Inflammatory status and comorbidities: the presence of laryngeal or tracheal reactivity, as proven by the presence of erythematous mucosa or edema, is a contraindication for proceeding to open neck surgery; the same policy should be followed if the child is suffering from unresolved concomitant cardiopulmonary disease, neurological impairment associated with pharyngolaryngeal discoordination and/or aspiration or uncontrolled gastroesophageal reflux disease.
3. Age and weight of the child: when an infant has no significant comorbidities, early intervention during the first months of life is appropriate (2). Thanks to the established use of magnification devices and the experience that has been accrued in tertiary referral centers for the treatment of infants weighing <10 kg (29), the weight criterion no longer needs to be considered an absolute contraindication. As we are dealing with growing tracheostomized children, it must always be kept in mind that a delay would ultimately affect both communication learning (30) and the mortality rate associated with the tracheostomy tube.

### Patient Management

Currently available treatments can be summarized as including endoscopic procedures, open neck laryngo-tracheal reconstruction (single stage/double stage PCTR/LTR) and the use of non-surgical devices, such as continuous positive airway pressure (C-PAP) therapy. Patients with mild stenosis (grade I-II) often have intermittent stridor associated with respiratory tract infection, but tracheostomy is not usually needed. In this scenario the airway is not severely compromised and in most cases no treatment is necessary, especially if the stenosis is congenital and therefore it is expected to enlarge as the child grows.

When dealing with a symptomatic child requiring treatment, once the whole diagnostic algorithm has been followed, the decision-making should be discussed among the multidisciplinary airway team in order to reach a consensus for the best timing and type of surgery.

Monnier proposed a list of queries that the laryngotracheal stenosis board team should address so as to make the best treatment choice (2):

1. What type of surgery should be appropriate?
  a. Endoscopic, LTR, PCTR or extended PCTR?
  b. Which type of grafting if needed?
2. Single-stage or double-stage surgery?
  a. With or without stenting?
  b. Need for intensive care unit?
3. What type and risk of complications to expect?
  a. PCTR: anastomotic dehiscence and/or RLN injury
  b. LTR: high recurrence rate for grade III-IV subglottic stenosis
4. Which kind of results can be predicted about airway, voice and deglutition?
5. Have all relevant comorbidities been studied?
  a. GOR disease?
  b. Neurologic, pulmonary and cardiac diseases?
  c. Nutritional status?
  d. Airway infection or contamination?
6. Is surgical timing appropriate?
  a. Maturity of the stenosis?
  b. Patient's age and comorbidities?
7. Does the theoretically best surgical procedure fit with the child's comorbidities or other airway abnormalities?

**Figure 9:**
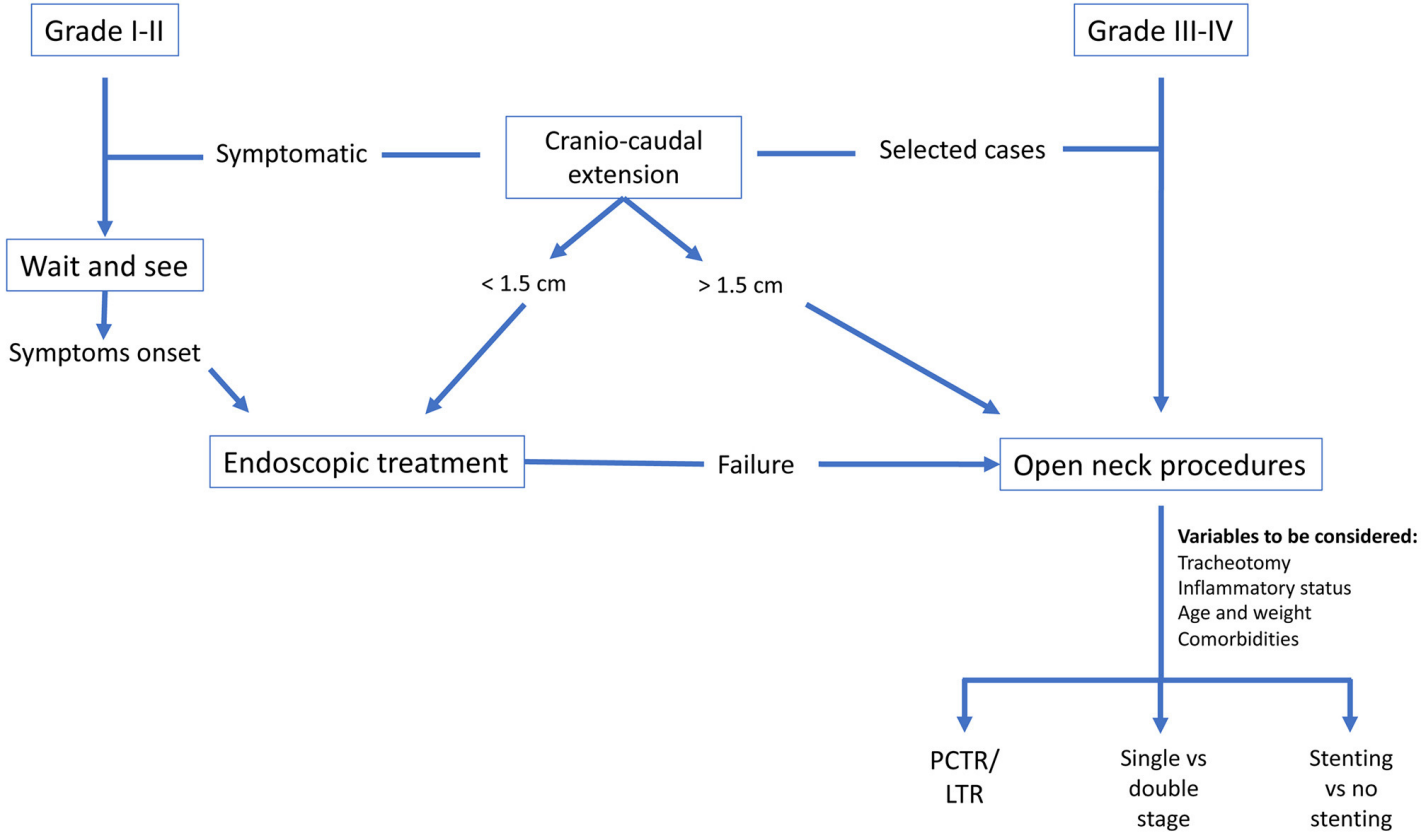
Decision-making flow-chart.

We propose our decision-making flow chart (Figure 9) since there is a broad number of treatment options and it is the task of the multidisciplinary team to establish which is the best for each individual patient while keeping in mind the aforementioned issues. Moreover, choosing the correct treatment modality at the very beginning is of paramount importance, otherwise the success of the entire treatment could be compromised.

## Author Contributions

Material preparation and literature search were performed by MF, FMa, and FMi. The first draft of the manuscript was written by MF, FMa, and FMi. GP and FC reviewed the manuscript. All authors contributed to the study conception and design, read, and approved the final manuscript.

### Conflict of Interest

The authors declare that the research was conducted in the absence of any commercial or financial relationships that could be construed as a potential conflict of interest.
